# Design of Unattended Rain and Snow Protection Device for Total Station Based on D-S Evidence Fusion

**DOI:** 10.3390/s26082327

**Published:** 2026-04-09

**Authors:** Liangquan Jia, Yong Liu, Guangzeng Du, Xinxin Li, Zhikang Wang, Yujie Lu, Zhibin Zhang

**Affiliations:** 1 School of Information Engineering, Huzhou University, Huzhou 313000, China; 2023388314@stu.zjhu.edu.cn (Y.L.); 2024388111@stu.zjhu.edu.cn (G.D.); 2024388208@stu.zjhu.edu.cn (X.L.); 02896@zjhu.edu.cn (Y.L.); 2 Shanghai Astronomical Observatory, Chinese Academy of Sciences, Shanghai 200030, China; wangzhikang@shao.ac.cn; 3 University of Chinese Academy of Sciences, Beijing 100049, China

**Keywords:** D-S evidence theory, multi-source information fusion, protection system, state machine, weight adjustment mechanism

## Abstract

Aiming at the rain protection problem of outdoor precision instruments such as total stations, this paper designs an environmentally adaptive intelligent protection system based on D-S evidence fusion and state machine control. In terms of mechanical structure, the protective cover is designed as a sinking type, which not only improves the safety of the equipment but also avoids the shielding problem of the working surface compared with the traditional upward-lifting structure. The system collects data from multi-source meteorological sensors and uses D-S evidence theory for fusion decision-making. To alleviate decision conflicts in high-conflict scenarios, a conflict-guided dynamic weight adjustment strategy is introduced. Combined with a dual-layer finite state machine, the system realizes coordinated control between environmental perception and protection actions and can activate protection within 30 s under severe weather. Simulation results show that the improved method increases the response speed by 41.9–62.5% compared with traditional D-S fusion in weather transition conditions. In a 28-day field test, the system achieves a daily protection success rate of 96.4% and 100% reliability in critical weather transitions. The proposed system can provide reliable support for the all-weather safe operation of field precision measurement equipment.

## 1. Introduction

Total stations, as core precision measuring instruments in modern engineering surveying, astronomical observation, and field geological monitoring, are increasingly deployed in unattended outdoor scenarios (such as very long baseline interferometry, VLBI observatories) to achieve long-term continuous automatic measurement [1,2]. For these scenarios, the measurement accuracy of the total station directly determines the validity of observation results, and long-term stable operation without manual intervention is a rigid core requirement. Although mainstream mid-to-high-end total stations (such as Leica TM60) are equipped with IP65-rated waterproof and dustproof enclosures, this passive protection can only resist low-pressure water spray and short-term moderate rainfall in accordance with IEC standard specifications. It cannot solve the core pain points in long-term unattended operation: rain and snow easily contaminate optical lenses and angle-measuring prisms, causing light refraction and a sharp decline in measurement accuracy; long-term exposure to humid environments may also lead to water vapor penetration and internal circuit oxidation, thereby permanently damaging precision components and shortening service life.

At present, outdoor total station protection mainly relies on manual guarding and passive rain covers. Manual intervention has defects such as slow response, high labor costs, and inability to cope with sudden weather changes, making it difficult to meet the needs of unattended measurement. Most existing unattended protection systems use a single rainfall sensor for environmental perception, which is susceptible to dew, water splashing, and inherent sensor defects, resulting in high false alarm rates and response delays [3]. Therefore, it is urgent to develop an environment-adaptive intelligent rain and snow protection system with multi-source sensing, fast decision-making, and reliable execution.

Multi-source information fusion and intelligent decision-making technology provide key technical support for environment-adaptive protection of outdoor precision instruments [4]. By fusing complementary information from heterogeneous sensors, such methods reduce the uncertainty and interference of single-source data, thereby improving the accuracy and reliability of environmental state judgment. Mainstream fusion methods include Bayesian inference, fuzzy logic, neural networks, and D-S evidence theory. In recent years, researchers have proposed a variety of advanced machine learning schemes to achieve robust environmental perception, such as enhanced Bayesian dynamic models and uncertainty-aware sparse Bayesian learning methods, which show good application prospects in processing complex time-varying monitoring data [5,6]. Despite their excellent data processing performance, these Bayesian-based machine learning methods still rely on complex statistical inference and high-dimensional matrix operations, which bring excessive computational burden to the resource-constrained STM32 embedded platform, making it difficult to meet the millisecond-level real-time requirements of this protection scenario.

For the STM32F407 embedded platform used in this study, the computational complexity, memory usage, and real-time performance of the fusion algorithm directly determine its engineering feasibility. Bayesian inference requires accurate prior probabilities and involves complex matrix operations with high computational cost, making it unsuitable for resource-constrained embedded platforms [7,8]. Fuzzy logic relies on manually designed fuzzy rules and has poor generalization ability in complex and variable outdoor meteorological environments [9,10]. Neural networks have strong nonlinear fitting ability but require a large amount of labeled training data. Their convolution operations have high requirements for computing power, making it difficult to meet the real-time perception needs of outdoor embedded systems [11,12].

In contrast, D-S evidence theory does not require prior probability information. Its core operations only involve set operations and credibility synthesis with low computational cost. It can effectively process uncertain and conflicting data from multi-source sensors and is especially suitable for environmental perception and decision-making in embedded protection systems [13,14,15].

However, the application of D-S evidence theory in the field of outdoor precision instrument protection is still in the development stage, with two key problems remaining. First, traditional D-S evidence theory tends to produce counterintuitive fusion results (such as the Zadeh paradox) when dealing with highly conflicting evidence [16]. In actual rainfall perception, different sensors have inherent differences in response characteristics: contact sensors recover slowly after rain stops, while optical sensors have low sensitivity to light rain. Such differences lead to severe evidence conflicts during weather transitions, resulting in decision delays or misjudgments.

Although many improved D-S methods have been proposed to address this issue, most of them focus on theoretical conflict resolution without realizing adaptive weight adjustment tailored to the response characteristics of rainfall sensors and the requirements of weather transition scenarios. For instance, in 2017, Li et al. combined a similarity matrix with a modified Gini coefficient to calculate the credibility of each piece of evidence [17]. They used this credibility as the weight to correct the original evidence, so as to mitigate the negative impact of conflicting evidence on fusion results. However, this method is designed to resolve the complete conflict paradox based on multi-sensor data, and its solution involves complex matrix operations, which may lead to insufficient real-time performance when deployed on embedded platforms such as the STM32 microcontroller. In 2024, Du et al. proposed to alleviate evidence conflicts by adjusting the dimension of the basic probability assignment (BPA) and normalized the mass function m(A) via an exponential function to narrow the gap between different m(A) values [18]. Nevertheless, this method still fails to handle the complete conflict paradox scenario with only two sensors, which is the most common case in the low-cost rainfall perception system for outdoor instrument protection.

Second, existing total station protection systems lack an efficient execution architecture matching multi-source fusion decisions. A single finite state machine structure is difficult to coordinate environmental perception and protection actions and cannot fully meet safety requirements such as overcurrent protection and emergency stop, which restricts the engineering practicability of the entire system [19,20].

To fill the above research gaps, this paper proposes three targeted improvements from the three dimensions: fusion algorithm, control architecture, and hardware implementation.

First, a dynamic weighted fusion mechanism for conflict resolution is proposed. The dynamic weight adjustment rule is fully customized based on the inherent response characteristics of contact and optical rainfall sensors. When the evidence is in a state of complete conflict, the mechanism temporarily jumps out of the classical D-S fusion framework. Considering that the sensor response to weather changes has an inherent delay, the conflict coefficient K will gradually decrease over time, shifting from a complete conflict state where fusion cannot be validly performed to a low-conflict state that supports reliable fusion calculation, after which the system control is handed over to the classical D-S evidence theory. This method not only improves the system response speed under high conflict conditions, but it also retains the stability advantage of D-S evidence fusion in low-conflict scenarios.

Second, a dual-layer finite state machine (FSM) architecture is designed to separate the policy decision layer and the execution control layer. The policy layer completes environmental state judgment and protection strategy generation based on fusion results, and the execution layer independently implements protection action control and safety monitoring such as overcurrent protection, realizing intelligent coordination between environmental perception and actuators.

Third, the hardware structure and drive strategy are optimized for outdoor long-term operation. Compared with common protection devices, a sinking design is adopted to reduce horizontal occlusion of the total station lens. A soft-start control strategy is adopted for the linear actuator of the protection device to optimize output current characteristics, reduce electrical stress on the drive circuit, and improve the long-term operation stability of the system in outdoor environments.

Simulation results show that the proposed dynamic weighted fusion mechanism improves the system response speed by 41.9~62.5% compared with the traditional D-S method during typical weather transition scenarios. A 28-day continuous field test conducted at the VLBI Observatory of Shanghai Astronomical Observatory shows that the system achieves a daily protection success rate of 96.4% and 100% reliability in key weather transition scenarios, which can provide stable and reliable all-weather protection for unattended outdoor total stations.

## 2. Multi-Source Environmental Perception Fusion and State Machine Decision-Making

**Figure 1:**
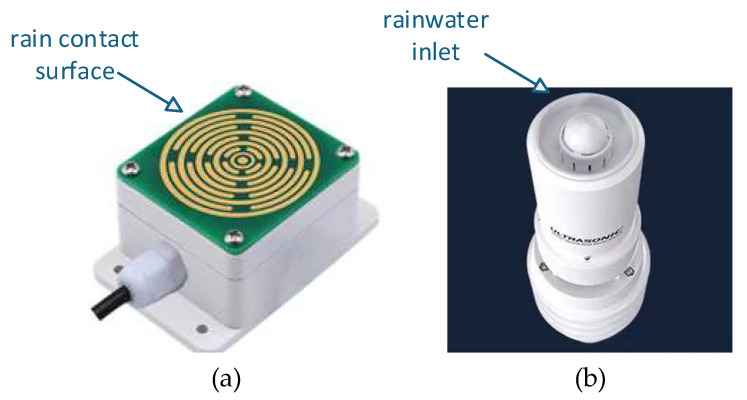
Photograph of the sensor prototype. (**a**) Contact rain and snow sensor; (**b**) optical rain sensor.

This device adopts a contact-type rain sensor and a digital optical tipping bucket rain sensor for rainfall detection. The two sensors exhibit complementary response characteristics: the contact-type sensor has high sensitivity to light rain and can quickly capture initial rainfall signals, but it is prone to persistent false detection of rainfall due to surface water accumulation after rain stops, with a recovery delay of 2–4 h. The tipping bucket rain sensor is insensitive to light rain and outputs valid signals only when the rainfall intensity reaches a certain level; however, it responds rapidly to rainfall fading and disappearance without recovery delay. The physical object is shown in Figure 1.

If the AND logic of dual sensors is adopted for rainfall decision-making (actions are executed only when both sensors confirm rainfall or no rainfall), serious response lag will occur due to inherent sensor drawbacks: during rainfall, the system has to wait for the tipping bucket sensor to trigger, thus missing the early protection window; after rain stops, the system has to wait for water evaporation on the contact-type sensor, so the protection device cannot be retracted in time, which affects the normal measurement of the total station.

Therefore, this paper introduces the D-S evidence theory to implement dual-sensor data fusion. By making full use of their complementary characteristics, the system relies on the high sensitivity of the contact-type sensor during rainfall and the fast recovery characteristic of the tipping bucket sensor after rain, so as to overcome the lag problem of AND logic decision-making. Meanwhile, it reduces the misjudgment risk of a single sensor and provides accurate and real-time rainfall judgment for the protection device.

### 2.1. Meteorological Data Fusion Based on D-S Evidence Theory

D-S evidence theory, also known as Dempster–Shafer theory, is widely used in information fusion, decision analysis, pattern recognition, and other fields. For rainfall detection in the total station protection scenario, the frame of discernment for multi-source meteorological information fusion is defined as $\theta \;=\;\{Q_{1},Q_{2},Q_{3}\}$, where $Q_{1}$, $Q_{2}$ and $Q_{3}$ represent rainy, non-rainy, and uncertain weather conditions, respectively. The evidence set is defined as $\varphi =\{A_{1},A_{2}\}$, where $A_{1}$ and $A_{2}$ correspond to the contact rain and snow sensor and the digital optical tipping bucket rain sensor—the two most commonly used rainfall detection sensors in the industrial field. The selection of the dual-sensor evidence set is based on their complementary response characteristics in outdoor rainfall detection, which can cover the full rainfall perception scenario from light rain to heavy rain and from rainfall onset to cessation, and avoid the perception blind zone caused by a single sensor. The basic probability assignment function $\mathrm{m:}\;2^{\theta }\to [0,1]$ is constructed to map the sensor’s physical detection signal to the belief degree of the discernment frame elements, and it satisfies the basic constraints given in Equation (1).

(1) $$\left\{\begin{array}{c} m(\emptyset )=0\\ \sum_{Q_{i}\subset \theta }m(A_{j}(Q_{i}))=1 \end{array}\right.$$

where m is the basic probability assignment function, and its output is referred to as the basic probability assignment (BPA), which can transform feature values into belief degrees. Here, ∅ denotes the empty set, and $m(A_{j}(Q_{i}))$ represents the support probability of the j-th sensor evidence for the i-th weather state in the discernment frame. The introduction of the uncertain category $Q_{3}$ is for the engineering practicality of outdoor unattended detection: the contact sensor is susceptible to false triggers by dew, bird droppings, and water splashing, while the optical sensor has a response delay for light rain; in such ambiguous perception scenarios, setting the uncertain category can avoid blind decision-making of the protection system. This design effectively reduces the misoperation rate of the protection device and improves the reliability of decision-making in complex outdoor environments.

Considering the inherent response characteristics and outdoor environmental interference factors of the two selected sensors, the contact sensor operates based on surface wetness detection, featuring fast response, but it is prone to false alarms caused by dew, water splashing, and bird droppings. Its BPA for the rainy state $Q_{1}$ is directly determined by the normalized wetness detection value of the sensor and the ambient humidity interference, as shown in Equation (2). The uncertainty probability of the contact sensor for $Q_{3}$ is set as a fixed low value to characterize the inherent detection uncertainty of the sensor, and the same uncertainty probability is assigned to the optical sensor to ensure the consistency of evidence uncertainty characterization, as shown in Equation (3).

(2) $$m(A_{1}(Q_{1}))=S_{wet},$$

(3) $$m(A_{1}(Q_{3}))=m(A_{2}(Q_{3}))=0.05,$$

In Formula (2), $S_{wet}$ is the normalized wetness value of the contact sensor, which is obtained by normalizing the resistance change signal of the sensor’s surface wetness detection module to the range of [0, 1]—the value is 1 when the sensor surface is completely wetted by water, and 0 when the sensor surface is completely dry, which directly reflects the wetness state detected by the sensor.

The contact rain sensor incorporates a constant-temperature heating structure, maintaining its surface temperature sufficiently high to eliminate condensation under all practical outdoor humid conditions. Since dew-induced interference is physically absent, no additional humidity correction is required. According to the basic constraint of the BPA function in Formula (1), the BPA of the contact sensor for the non-rainy state $Q_{2}$ is derived as:

(4) $$m(A_{1}(Q_{2}))\;=\;1\;-\;m(A_{1}(Q_{1}))\;-\;m(A_{1}(Q_{3})),$$

The optical rain gauge realizes rainfall detection by collecting the number of tipping bucket turns caused by rainwater accumulation, which requires rain accumulation resulting in a delayed response but high confirmation reliability for effective rainfall. Its BPA for the rainy state $Q_{1}$ is determined by the instantaneous rain intensity r (unit: mL/min), as shown in Equation (5).

(5) $$m(A_{2}(Q_{1}))=1-\lambda ∙\exp\left(-\frac{r}{r_{0}}\right),$$

where $r_{0}=0.2\;\mathrm{mL/min}$ is the characteristic rain intensity, which is the minimum effective rainfall intensity that the mainstream industrial optical tipping bucket rain sensor can stably detect. When the rainfall intensity r <0.2 mL/min, the optical sensor cannot output a stable rainfall signal, and the BPA for the rainy state $Q_{1}$ decreases rapidly; when r ≥0.2 mL/min, the BPA for the rainy state $Q_{1}$ approaches 1, which accurately characterizes the detection characteristics of the optical sensor that is insensitive to light rain and highly reliable for moderate and heavy rain. Similarly, the BPA of the optical sensor for the non-rainy state $Q_{2}$ is derived as:

(6) $$m(A_{2}(Q_{2}))\;=\;1\;-\;m(A_{2}(Q_{1}))\;-\;m(A_{2}(Q_{3})),$$

For evidence source fusion in D-S theory, the standard Dempster combination rule is adopted, as shown in Equations (7) and (8). K is the conflict coefficient used to describe the degree of conflict between evidence. A larger value indicates higher evidence conflict, meaning the decision result after fusion is unsatisfactory. The fusion of highly conflicting evidence may lead to counterintuitive results, such as the Zadeh paradox, which is the key problem to be solved in this paper.

(7) $$K=\sum_{B\cap C\;=\;\emptyset }m_{1}(B)m_{2}(C),$$

(8) $$(m_{1}⊕m_{2})(Q_{1})=\frac{\sum_{B\cap C=A}\;m_{1}(B)m_{2}(C)}{1-K},$$

where $m_{1}$ and $m_{2}$ represent the BPA functions of the contact sensor $A_{1}$ and the optical sensor $A_{2}$, respectively; A,B,C are arbitrary subsets of the discernment frame θ, and B∩C = ∅ means the two subsets are mutually exclusive. The conflict coefficient K in Formula (6) is the sum of the product of BPAs of all mutually exclusive subsets of the two sensors, which can fully characterize the overall conflict degree between the two sensor evidences in the rainfall detection scenario. Formula (7) is the standard Dempster combination rule, which normalizes the fusion result by the factor 1 − K to meet the basic constraint of the BPA function.

### 2.2. Evidence Conflict Resolution Mechanism Based on Adaptive Weighting

The traditional D-S evidence theory has certain limitations: when there is high conflict between evidence [14], it may lead to paradoxes, and the system may fail to respond quickly in some cases. In practical applications, the contact sensor and optical tipping bucket sensor have inherent complementary response characteristics (the contact sensor is sensitive to light rain but has post-rain recovery delay; the optical sensor is insensitive to light rain but responds quickly to rain cessation), which easily leads to high conflict of perception evidence during weather transition stages (sunny-to-rainy/rainy-to-sunny). Such conflicts will cause decision delay or misjudgment of the protection system, so it is necessary to optimize and handle potential evidence conflicts.

To address the above issues, this study proposes a conflict-oriented adaptive weight adjustment model. By real-time evaluating the degree of conflict between evidence and the change trend of rainfall state, the model dynamically adjusts the fusion weights of the two sensors. Thus, while retaining the advantages of the D-S theory framework, it enhances the decision-making efficiency and reliability of the system in critical weather transition scenarios.

First, a rainfall state trend judgment function Tr is constructed to identify the type of weather transition, as shown in Equation (9). The trend judgment result serves as the core basis for determining the weight adjustment strategy.

(9) $$Tr=sign(\Delta r+\Delta S_{wet}),$$

where sign() is the sign function; Δr = r(t) − r(t − 1) is the rainfall intensity change amount of the optical sensor at current moment t relative to previous moment t − 1 (unit: mL/min), reflecting the variation trend of effective rainfall intensity; $\Delta Swet\;=\;S_{wet}(t)\;-\;S_{wet}(t\;-\;1)$ is the wetness state change amount of the contact sensor at current moment t relative to previous moment t − 1, reflecting the variation trend of sensor surface wetness. The judgment rules of Tr are as follows:

1. When Tr > 0: At least one sensor detects an increasing rainfall tendency (the optical sensor detects rising rainfall intensity, or the contact sensor detects increasing surface wetness), indicating a potential sunny-to-rainy transition;
2. When Tr < 0: At least one sensor detects a decreasing rainfall tendency (the optical sensor detects dropping rainfall intensity, or the contact sensor detects decreasing surface wetness), indicating a potential rainy-to-sunny transition;
3. When Tr = 0: The detection states of both sensors have no obvious change, and the system maintains the initial equal weight fusion strategy.

When the conflict coefficient K ≥ 0.7 (the fusion strategy switching threshold determined by theoretical derivation and simulation optimization), the system triggers the adaptive weight adjustment mechanism, and the specific weight adjustment strategy is determined according to the trend judgment result Tr.

#### 2.2.1. Sunny-to-Rainy Conflict

At the initial stage of rainfall, the contact sensor responds immediately to light rain and outputs a wet signal due to its high sensitivity, but the optical tipping bucket sensor requires a certain amount of rain accumulation to output a stable rainfall signal, resulting in high conflict between the two sensors’ evidence. To give full play to the advantage of the contact sensor in early rainfall detection, the system reduces the fusion weight of the optical sensor and increases the weight of the contact sensor. The time decay weight function of the optical sensor is shown in Equation (10):

(10) $$\omega_{o}(t)\;={\;\omega }_{base}\exp\left(-\frac{t\;-\;t_{c}}{T_{e}}\right),$$

where $\omega_{o}(t)$ is the fusion weight of the optical sensor at current moment t; $\omega_{base\;}=0.5$ is the base weight, and the two sensors are initialized with equal weight under the normalization constraint that the sum of weights is 1; $t_{c}$ is the start time of evidence conflict (the moment when K ≥0.7 is first detected); $T_{e}=30$ s is the expected response time of the optical sensor, calibrated according to the rainwater accumulation delay characteristic of mainstream industrial optical tipping bucket sensors (the sensor can stably output rainfall signals within 30 s after rainfall reaches effective intensity).

#### 2.2.2. Rainy-to-Sunny Conflict

When rainfall ceases, the optical sensor immediately detects the disappearance of rainfall intensity and outputs a non-rainy signal, but the contact sensor still maintains a wet state due to surface water accumulation, resulting in high conflict between the two sensors’ evidence. To give full play to the advantage of the optical sensor in rain cessation detection, the system reduces the fusion weight of the contact sensor and increases the weight of the optical sensor. The time decay weight function of the contact sensor is shown in Equation (11):

(11) $$\omega_{c}(t)\;=\;\omega_{base}\exp\left(-\frac{t\;-\;t_{c}}{T_{d}}\right),$$

where $\omega_{c}(t)$ is the fusion weight of the contact sensor at current moment t; $T_{d}\;=\;1800$ s (30 min) is the natural drying time of the contact sensor, determined according to actual outdoor environmental test data (surface water of the contact sensor can be naturally dried within 30 min under normal temperature and wind conditions). Other parameters are consistent with Formula (10). The fusion weight of the other sensor is determined by the normalization constraint (sum of weights equals 1): $\omega_{c}(t)\;=\;1\;-\;\omega_{o}(t)$ (sunny-to-rainy conflict); $\omega_{o}(t)\;=\;1\;-\;\omega_{c}(t)$ (rainy-to-sunny conflict).

When the conflicting sensor completes its response (the optical sensor outputs a stable rainfall signal with r ≥ 0.2 mL/min in a sunny-to-rainy conflict, or the contact sensor outputs a dry signal with Swet = 0 in a rainy-to-sunny conflict), the fusion weights of the two sensors are restored to the base weight $\omega_{base}\;=\;0.5$ simultaneously, and the system switches back to the standard Dempster combination rule for evidence fusion. This design ensures that the system can dynamically adapt to the response characteristics of different sensors in different weather transition stages, effectively resolving evidence conflict and improving decision-making timeliness.

### 2.3. Weighted Fusion and Simulation

In this rainfall perception system, the conflict coefficient K exhibits an obvious step-like surge characteristic: immediately once the two sensors produce highly inconsistent outputs during weather transitions, K will rapidly rise from the low-conflict interval (below 0.3) to above 0.9 within a single sampling cycle, representing a typical strong conflict condition. Theoretical derivation indicates that traditional D-S fusion enters an unstable region when K > 0.8, where decision paradoxes (e.g., Zadeh paradox) are prone to occur. Further simulation-based threshold sensitivity verification shows that, for threshold values in the range of 0.6 to 0.8, the adjustment of the threshold only has a weak impact on the switching timing of the fusion strategy and will not change the final environmental state judgment result or the reliability of the protection action. To accurately and reliably detect such severe conflicts in a timely manner and activate the weighted fusion rule before the system enters the unstable interval, K = 0.7 is employed as the fusion strategy switching threshold; this threshold provides a clear and robust detection boundary with sufficient safety margin, enabling timely conflict identification while avoiding both premature switching and decision lag.

The system adopts an adaptive dual-mode fusion strategy based on the conflict coefficient K. When K < 0.7 (low evidence conflict), the two sensors provide consistent environmental perception information, and the system adopts the standard Dempster combination rule (Equations (5) and (6) in Section 2.1) for evidence fusion. This ensures the full utilization of complementary sensor information and maintains high decision accuracy. When K ≥ 0.7 (high evidence conflict), the sensor data exhibit significant inconsistency (typically occurring in weather transition stages), and the system switches to the conflict-oriented adaptive weighted fusion method proposed in Section 2.2. The fused basic probability assignment (BPA) for each state in the discernment frame is calculated as follows:

(12) $$m_{f}(Q_{i})\;=\;\omega (t)\cdot m_{1}(Q_{i})\;+\;(1\;-\;\omega (t))\cdot m_{2}(Q_{i}),$$

**Figure 2:**
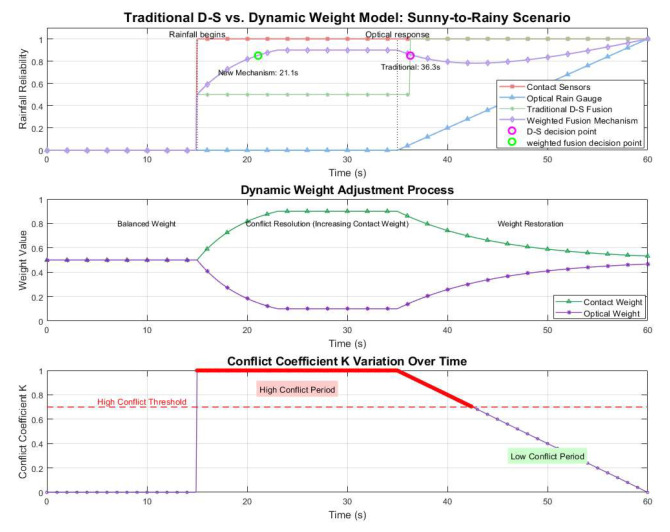
Simulation results of sunny-to-rainy transitions.

To verify the effectiveness of the proposed conflict-oriented adaptive weight adjustment model, simulation experiments were conducted on the MATLAB R2023b platform in this section, and the performance of the traditional D-S fusion method and the new model was compared and analyzed. Figure 2 presents the simulation results under the sunny-to-rainy transition scenario, including the sensor data, fusion results of the traditional D-S method, fusion results of the new model, and the variation curve of the conflict coefficient K. It can be observed that when rainfall starts at t = 15 s, the contact sensor responds immediately, while the optical rain gauge begins to output signals at t = 35 s due to its inherent delay characteristic. The traditional D-S fusion exhibits output oscillations during the conflict period (15–35 s). Assuming the decision threshold is set at 85%, the decision-making of the traditional D-S fusion is delayed until 36.3 s; in contrast, by reducing the weight of the optical sensor, the new model reaches the decision threshold at 21.1 s, with the response speed improved by 41.9%.

**Figure 3:**
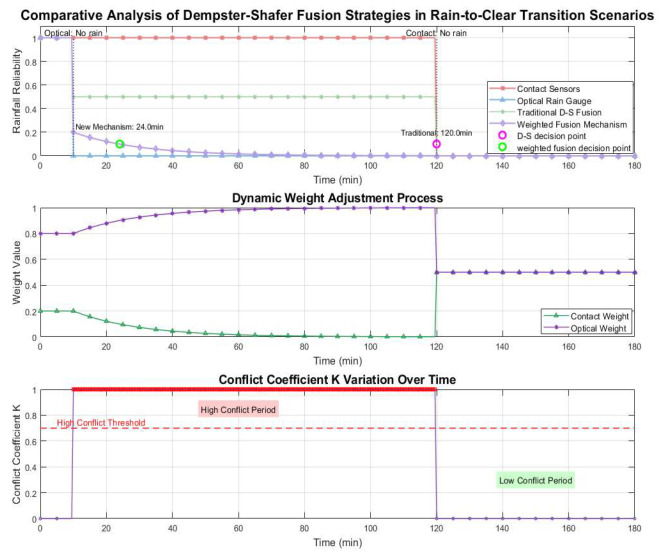
Simulation results of rainy-to-sunny transitions.

Figure 3 presents the simulation results under the rainy-to-sunny transition scenario, where the drying process of the contact sensor surface is assumed to occur from the 10th minute to the 120th minute. The decision-making of the traditional D-S fusion is delayed until the 120th minute, while the conflict-oriented adaptive weight adjustment model reaches the decision threshold at the 45th minute, resulting in a 62.5% improvement in response speed.

### 2.4. Autonomous Decision-Making Based on FSM

**Figure 4:**
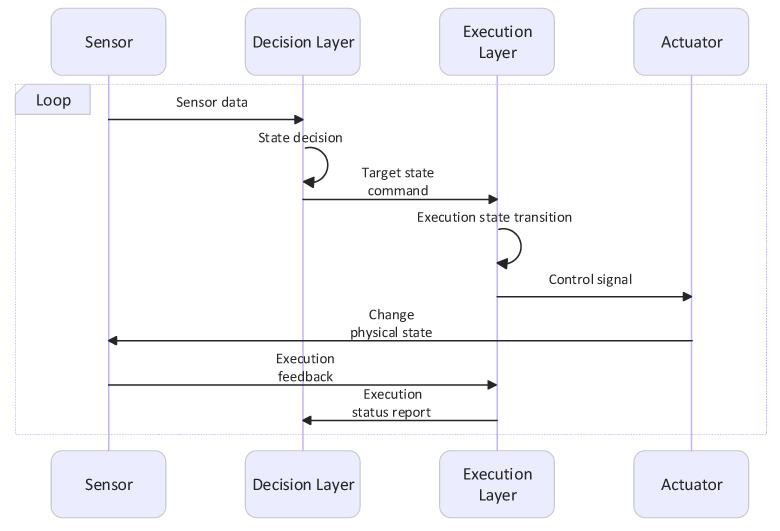
State interaction timing diagram.

The intelligent protection system adopts a finite state machine (FSM) as its core decision-making architecture [15,16]. Its state set *Q* includes idle, cooling, rising, falling, pause, emergency stop, and other combined states. However, using only one state machine to handle state transitions and execution is extremely difficult. Therefore, the system is designed with a dual-state machine architecture that separates policy decision-making from execution control. The policy layer focuses on environmental decision-making, while the execution layer can independently implement safety monitoring functions such as overcurrent protection. The state interaction timing sequence is shown in Figure 4.

## 3. Design of the Intelligent Protection Device

This intelligent protection system adopts the STM32F407 series microcontroller (specifically the STM32F407VET6 model from STMicroelectronics) as its core processing unit. The STM32F4 platform is selected for its excellent suitability for the system’s functional requirements: equipped with a Cortex-M4 core integrated with a single-precision Floating Point Unit (FPU), it can efficiently handle the floating-point operations required by the D-S evidence theory algorithm, including mass function calculation, conflict coefficient computation, and adaptive weight attenuation, ensuring the real-time performance of data fusion. Meanwhile, the STM32F407VET6 is rich in peripheral resources, with sufficient UART and RS485 interfaces to meet the communication needs of multi-sensor data acquisition and LoRa module connection. Its 168 MHz main frequency fully satisfies the requirements of state transition in the dual-layer finite state machine and the development of a simple user interface (UI), guaranteeing the stable and efficient operation of the control system. During full-load operation, the controller maintains an extremely low resource occupancy, with Flash usage at approximately 42.6 KB and SRAM usage at about 3.08 KB, ensuring sufficient hardware redundancy for long-term stable operation.

**Figure 5:**
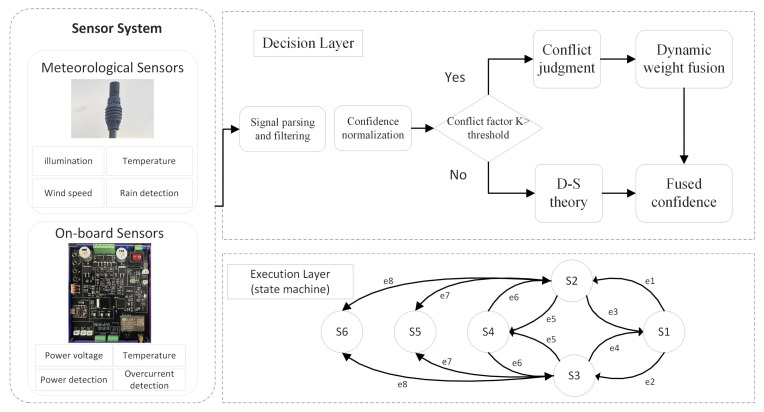
Overall structure diagram.

The microcontroller is responsible for collecting data from multi-source environmental sensors, deploying the D-S evidence theory algorithm, and driving the dual-layer state machine. The output part drives the circuit through an H-bridge combined with PWM modulation to achieve precise control over the intelligent protection device. The system is also equipped with a LoRa module configured with spread spectrum modulation, featuring a long transmission distance of up to 5 km in open areas. It supports short-range wireless communication based on low-power wide-area networks and multi-node networking control. The overall structure is shown in Figure 5. The left side consists of a sensor network and a self-developed hardware control circuit: the sensor network acquires information such as illumination, temperature and humidity, wind speed and direction, and rainfall, while the on-board sensors mainly obtain data, including system power supply monitoring and output current.

After the microcontroller obtains meteorological sensor data via ADC or RS485 communication, the data will enter the decision-making layer based on the D-S theory, where data analysis is performed and corresponding execution actions are derived according to the analysis results. For instance, a protection decision will be triggered based on rainfall meteorological data. Finally, the execution unit carries out actions such as lifting protection, activating the buzzer alarm, and sending data to the host computer for a status update.

### 3.1. Mechanical Structure Design

**Figure 6:**
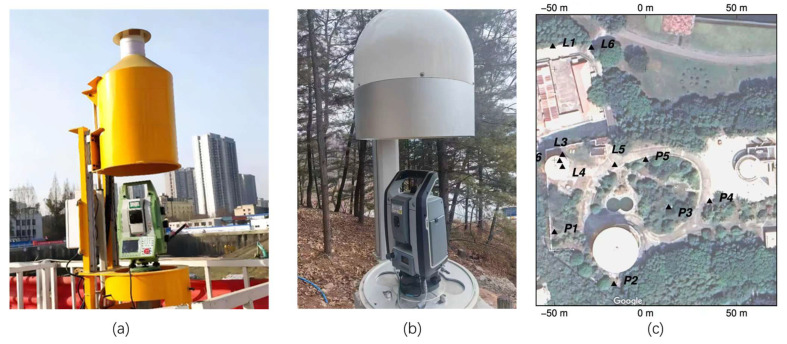
(**a**) QB350 protective device; (**b**) DT-BHZ-X1 protective device; (**c**) layout of protective devices.

Existing total station equipment mainly adopts a single-side supported lifting structure, such as the QB350 total station protective cover shown in Figure 6a and the DT-BHZ-X1 total station protective cover shown in Figure 6b. This structure suffers from significant visual obstruction on one side. Since a total of five devices are employed in this study, arranged as labeled P1–P5 in Figure 6c, and mutual monitoring between any two points is required, a high field of view is therefore demanded.

**Figure 7:**
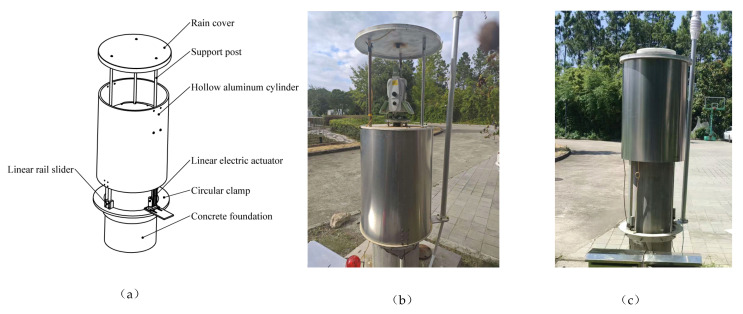
(**a**) Mechanical structure diagram of the model; (**b**) descending unprotected state; (**c**) ascending protected state.

The target protected in this study—the total station—is installed on a concrete pier 1200 mm above the ground. A metal mounting plate and a stainless-steel base are pre-embedded on the surface of the pier to fix the total station, and a GNSS antenna [21] also needs to be installed. Considering the requirement of low obstruction, the designed model of the device is shown in Figure 7a. The device is currently installed at the P4 point shown in Figure 6c.

The protective device investigated in this study is a liftable cylindrical shield. Its top part is a circular metal plate with a downward flange along the edge. Since its diameter is larger than that of the hollow cylindrical aluminum tube below, the two can interlock with each other to form a sealed, windproof and rainproof space. Three support rods with a diameter of 20 mm are arranged beneath to support the upper metal plate, while satisfying the requirement of low visual obstruction. A guide column system is installed at the lower part, which can effectively constrain the movement trajectory of the housing and prevent deflection or jamming during lifting. When rainfall is detected by the system, the linear actuator drives the aluminum tube to rise rapidly, forming a closed protective space as shown in Figure 7c. After the rainfall stops, the aluminum tube descends smoothly along the guide columns, returning the total station to its working state as shown in Figure 7b.

**Figure 8:**
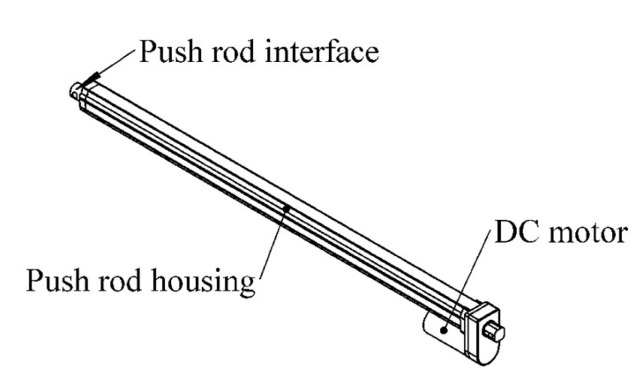
Electric actuator model.

The device adopts an electric linear push rod as the core driving component, which drives the cylindrical aluminum alloy housing to reciprocate up and down along multiple guide columns, so as to realize automatic protection for the total station on the top platform. The actuator model is shown in Figure 8.

This protective device is a lifting cylindrical shield, which adopts an electric linear actuator as the core actuator to drive the cylindrical aluminum alloy shell to reciprocate up and down along multiple guide pillars, realizing automatic protection for the total station on the top platform. The device is composed of a top metal cover plate, a cylindrical aluminum protective barrel, a linear actuator, and a base guide pillar system. The installed prototype is shown in Figure 7b. The aluminum alloy shell has the advantages of both light weight and structural strength, and the guide pillar system can effectively constrain the movement trajectory of the shell to avoid deflection or jamming during lifting. When the system detects rainfall, the linear actuator drives the aluminum barrel to rise quickly, forming a closed protective space; when the rainfall ends, the aluminum barrel descends smoothly along the guide pillars, returning the total station to an operational state.

### 3.2. State Machine Driven Design

Upon power-on, the system first performs an on-board self-test to initialize the STM32 microcontroller, multi-source meteorological sensors, motor drive circuits, LoRa communication module, and overcurrent protection circuit. It then transmits a status report to the host computer via the LoRa module, establishing a bidirectional communication link.

Once entering the main loop, the system continuously collects meteorological data and executes decision-making through the D-S evidence theory. In a low-conflict sunny scenario, when the conflict coefficient K of the D-S fusion is below the preset threshold and the decision result indicates sunny conditions, the system reads the limit switch status to determine the posture of the actuator. If the system is in a rising protection state, it triggers a descending recovery command; if it is in a descending idle state, the system maintains the idle status without actuator movement. In a low-conflict rainy scenario, the system checks the limit switch status: if the actuator is in a descending posture, it executes a rising protection command; if it is already in a rising protection state, the system maintains the current status.

To handle sensor conflicts during weather transitions, an adaptive weight attenuation strategy is implemented. When transitioning from sunny to rainy conditions, the weight of the optical non-contact raindrop sensor is attenuated over time; conversely, when transitioning from rainy to sunny conditions, the weight of the contact-type rain sensor is attenuated over time, thereby improving the system’s response speed.

**Figure 9:**
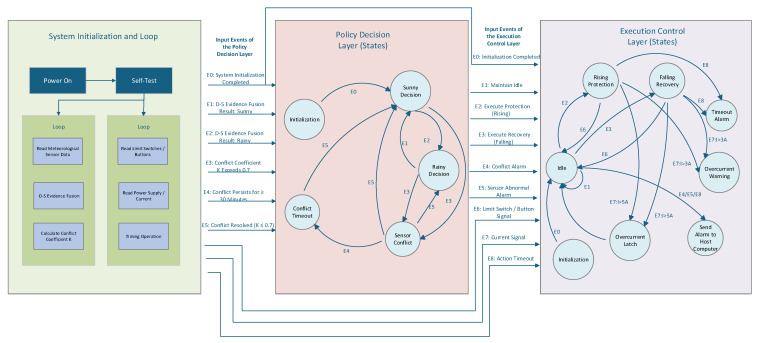
Dual-Layer FSM for Intelligent Protection System.

During the decision-making process, if the conflict coefficient K remains above the preset threshold for a continuous duration of 30 min, the system determines a sensor anomaly. Based on the design principle of “err on the side of protection rather than missing protection”, it forcibly executes the protection command and sends an alarm to the host computer, prompting manual inspection of the sensors to ensure system reliability. The dual-layer finite state machine and state transitions of the device operation are shown in Figure 9.

The system adopts a dual-layer finite state machine (FSM) architecture, which is divided into three core modules: the system initialization and main loop, policy decision layer, and execution control layer. The policy decision layer is responsible for weather state judgment and conflict handling, and outputs control commands to the execution layer; the execution control layer receives commands, drives the actuator, and implements fault protection and alarm functions, forming a closed-loop control system.

The policy decision layer takes the output of the data fusion module as input events, and realizes weather state decision and conflict handling through five states:

1. Initialization State:

After the system initialization is completed (triggered by event E0), the state transitions to the Sunny Decision state by default.

2. Sunny Decision State:

When the D-S fusion result is judged as sunny (event E1), the state remains; when the fusion result is judged as rainy (event E2), it transitions to the Rainy Decision state; when the conflict coefficient K exceeds 0.7 (event E3), it transitions to the Sensor Conflict state.

3. Rainy Decision State:

When the fusion result is judged as rainy, the state remains; when the fusion result is judged as sunny (event E1), it transitions back to the Sunny Decision state; when the conflict coefficient K exceeds 0.7 (event E3), it transitions to the Sensor Conflict state.

4. Sensor Conflict State:

When the conflict coefficient K is lower than the threshold (event E5, K ≤ 0.7), it transitions back to the corresponding sunny/rainy decision state according to the fusion result; when the conflict persists for ≥30 min (event E4), it transitions to the Conflict Timeout state.

5. Conflict Timeout State:

Based on the design principle of “prefer false protection to missing protection”, the system forcibly outputs a protection command and only transitions back to the Sunny Decision state after the conflict is resolved or manually reset (event E5).

The policy decision layer outputs control commands (maintain idle, execute protection, execute recovery, conflict alarm, sensor abnormal alarm) to the execution control layer according to the state transition results, realizing the closed-loop control of the system.

The execution control layer takes the control commands of the policy decision layer, limit switch signals, current signals and timing information as input events, and realizes actuator drive and fault protection through seven states:

1. Idle State:

After initialization is completed (event E0), the system enters the idle state by default. When receiving the “execute protection (rising)” command (event E2), it transitions to the Rising Protection state; when receiving the “execute recovery (falling)” command (event E3), it transitions to the Falling Recovery state; when receiving the “maintain idle” command (event E1), the state remains.

2. Rising Protection State:

After the system initialization is completed (triggered by event E0), the state transitions to the Sunny Decision state by default. The actuator executes the rising action, and when the upper limit switch is triggered (event E6), it transitions back to the Idle state; when the drive current I > 3A (event E7), it transitions to the Overcurrent Warning state; when the current I > 5A (event E7), it directly transitions to the Overcurrent Latch state.

3. Falling Recovery State:

After the system initialization is completed (triggered by event E0), the state transitions to the Sunny Decision state by default. The actuator executes the falling action and starts a 30 s timing. When the lower limit switch is triggered (event E6), it transitions back to the Idle state; when the action times out (event E8), it transitions to the Timeout Alarm state; when the drive current I > 3A (event E7), it transitions to the Overcurrent Warning state; when the current I > 5A (event E7), it directly transitions to the Overcurrent Latch state.

4. Overcurrent Warning State:

The system sends a lubrication maintenance alarm to the host computer and transitions back to the original running state after the current recovers.

5. Overcurrent Latch State:

The system immediately cuts off the drive circuit, locks the actuator, and only transitions back to the Idle state after manual reset.

6. Timeout Alarm State:

The system sends an action timeout alarm to the host computer and only transitions back to the Idle state after a manual reset.

7. Send Alarm to Host Computer State:

When receiving a conflict alarm, sensor abnormal alarm or timeout alarm (events E4/E5/E8), the system sends the corresponding alarm information to the upper computer through the LoRa module and then returns to the Idle state.

## 4. Experimental Setup and Results

### 4.1. Experimental Site and Test Scenarios

The experiment was conducted at the VLBI Observatory of the Shanghai Astronomical Observatory, Chinese Academy of Sciences, to simulate the actual working environment of precision measurement equipment in unstaffed outdoor conditions. Three categories of key test scenarios were designed to fully verify the system’s adaptability and reliability:

1. Sustained weather conditions

Including continuous sunny days and continuous rainy days, focusing on testing the system’s stability under constant meteorological conditions.

2. Variable weather conditions

Covering sunny-to-rainy and rainy-to-sunny transitions, aiming to evaluate the system’s response speed and decision accuracy during meteorological changes.

3. Output current conditions

Monitoring the real-time output current of the protection device to verify the safety and rationality of the actuation control strategy.

### 4.2. Output Current Monitoring

**Figure 10:**
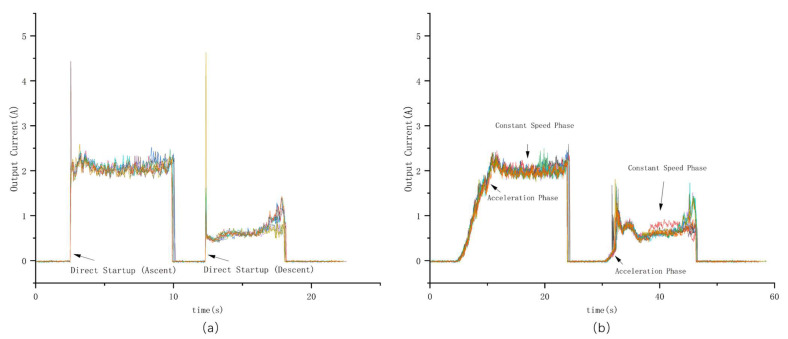
Comparison of Current Curves Between Direct Startup and Soft Startup for the Linear Actuator. (**a**) Superposition of 10 Current Curves for Direct Startup; (**b**) Superposition of 10 Current Curves for Soft Startup.

To evaluate the starting performance of the linear actuator drive circuit, this study tests the output current under direct startup and soft-start modes, with the experimental results shown in Figure 10. The experimental results show that direct startup causes the motor to reach its rated speed instantaneously, resulting in sharp current spikes, with peak currents as high as 4.5 A and 4.6 A during the ascending and descending processes, respectively. This not only imposes severe electrical stress on the power supply and drive chip but also increases the risk of false triggering of the overcurrent protection. In contrast, the soft-start design ensures a smooth acceleration of the motor speed, and the current curve exhibits distinct acceleration and constant speed phases, with the peak currents effectively suppressed to 2.5 A and 2.3 A, representing a reduction of more than 40%.

At a supply voltage of 24 V, the corresponding operating power is approximately 62.4 W, which is far below the rated power of the configured 240 W power supply, providing sufficient redundancy for long-term operation. Meanwhile, the system maintains an extremely low standby power consumption of less than 2 W during normal monitoring. To further ensure operational safety, an overcurrent alarm threshold is set at 3 A (corresponding to 72 W). If the actual driving current exceeds this threshold, it indicates potential mechanical aging or insufficient lubrication, allowing timely maintenance to be carried out. This optimization provides a reliable reference for setting the overcurrent protection threshold, reduces system power consumption, and enhances the long-term operational stability of the device in outdoor environments.

### 4.3. Test Conditions and Experimental Configuration

Based on the simulation and output current monitoring results, four key test trigger conditions were defined to standardize the experimental judgment criteria:

1. Emergency stop condition

The output current of the protection device exceeds 5 A.

2. Overcurrent condition

The output current exceeds 3 A.

3. Rain condition

Rainfall confidence from D-S multi-source meteorological information fusion ≥ 0.85, or rainfall confidence from the weight adjustment model ≥ 0.8.

4. Sustained weather conditions

Rainfall confidence supported by D-S multi-source fusion results ≤ 0.15, or rainfall confidence from the weight adjustment model ≤ 0.2.

The experimental device was installed on 6 May 2025, followed by a five-month long-term operation test. After optimizing the D-S evidence fusion algorithm, improving the weight adjustment model, and updating the system software, the test data from October 2025 were selected for formal analysis (to ensure data validity after algorithm iteration). The data collection period was from 1 October to 28 October 2025, with data recorded at 8:00 a.m. daily. Weather information was obtained from public meteorological datasets to provide ground truth for result verification.

### 4.4. Test Results and Data Analysis

**Figure 11:**
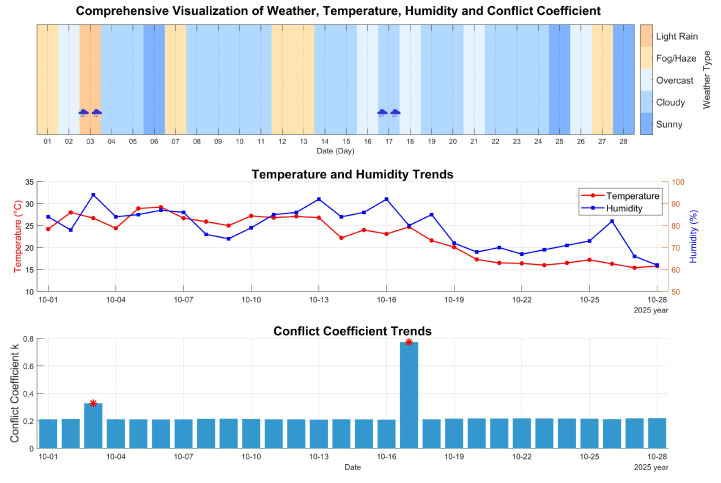
Coverage test results under daily scenarios.

As shown in Figure 11, the system maintained stable and reliable operation across all typical daily weather conditions observed during the 28-day field test, including sunny, cloudy, overcast, fog/haze, and light-to-moderate rainfall. To quantitatively evaluate the system’s performance, we conducted a detailed analysis of key metrics under different weather transition scenarios. In the conflict coefficient trend subplot, the asterisk “*” indicates that sensor conflict was triggered on that date. As can be seen, on 3 October 2025, rainfall occurred with a fusion rainfall confidence of 86.54%, and the system successfully triggered the lifting protection mechanism, verifying its sensitivity to effective rainfall signals. The only abnormal event occurred on 17 October 2025: under cloudy weather conditions, the protection device was falsely activated. Post-inspection confirmed that bird droppings on the contact sensor caused a false trigger. At this time, the conflict coefficient K rose sharply to 0.82, exceeding the switching threshold of 0.7. However, the current fusion strategy still assigned a relatively high weight to the affected contact sensor, leading to an incorrect decision. This false alarm not only verifies the effectiveness of the conflict detection mechanism but also provides clear guidance for subsequent improvements, such as introducing temporal consistency checks and multi-point distributed fusion to enhance the system’s anti-interference capability against non-meteorological disturbances.

Table 1 presents the detailed results of six groups of key weather transition tests, covering all typical outdoor working scenarios (cloudy → light rain, light rain → overcast, overcast → light rain, light rain → fog, etc.). To ensure stable and reliable actuation, the system adopted a soft-start control strategy for the linear actuator. As shown in the table, the output current during all protection/retraction actions was maintained below 3 A, with an average operating current of only 2.16 A. This current level is far below the overcurrent protection threshold (5 A), effectively avoiding false overcurrent triggers and reducing electrical stress on the drive circuit and power supply, significantly improving the long-term operational stability of the system. The core advantage of the proposed improved D-S fusion algorithm is its dynamic weight adjustment mechanism. During sunny-to-rainy transitions, the weight of the contact rain sensor dynamically increased from the initial value of 0.5 to a maximum of 0.932, enabling the system to detect rainfall rapidly and deploy the protective mechanism within 30 s. Conversely, during rainy-to-sunny transitions, the sensor weight decreased from 0.2 to a minimum of 0.128, ensuring timely retraction of the protection device and restoring the instrument to normal working condition without occlusion. All six transition tests were completed successfully, verifying that the system achieves full coverage of typical outdoor working scenarios and exhibits strong adaptive and dynamic adjustment capabilities.

Notably, the conflict coefficient K (as shown in Figure 11) exhibited an obvious step-change during weather transitions, rising from near 0 to above 0.8 in high-conflict scenarios. The threshold of 0.7 set in this study effectively triggered the weighted fusion rule before the system entered the unstable region (K > 0.8), avoiding decision paradoxes such as the Zadeh paradox, and ensuring the reliability of the fusion decision.

For concise presentation, weather condition labels are abbreviated as follows: C denotes Cloudy, O denotes Overcast, LR denotes Light rain, F denotes Fog, and the symbol → represents the transition from one weather state to another.

## 5. Discussion

The experimental results demonstrate that the proposed intelligent protection system achieves satisfactory recognition accuracy and reliable operation under complex outdoor meteorological conditions. By integrating a conflict-guided dynamic weight adjustment mechanism into the D-S evidence theory, the system effectively mitigates decision conflicts caused by inconsistent or abnormal sensor outputs, thereby improving the response speed to multi-sensor conflicts. A test accuracy of 96.4% in long-term field deployment validates the system’s practicality and robustness. During weather transitions, smooth weight adaptation enables the system to respond to rainfall events while avoiding frequent malfunctions triggered by weak meteorological signals.

Notably, the proposed system adopts a subsidence-type protective structure, which offers comparative advantages over traditional ascending-type protective devices (e.g., the QB350 and DT-BHZ-X1 systems). In ascending-type designs, the protective cover is lifted above the total station during non-protection periods, which may cause occlusion to the instrument’s horizontal and upward-looking working ranges and interfere with normal measurement. Additionally, placing the heavy cover at a higher position may subject the entire device to greater lateral force under high wind speeds, affecting structural stability.

In contrast, the subsidence-type structure sinks the protective cover and drive actuator to the lower part of the device in the non-protection state, which helps minimize horizontal obstruction to the total station’s working area. Meanwhile, the sunken protective housing is integrated with the cement foundation. In the event of mechanical failure, this integrated design reduces the risk of direct damage to precision instruments such as the total station. This structural design effectively reduces the impact on the instrument’s working range and improves field operation convenience.

A single false detection incident occurred due to bird droppings interfering with the contact sensor, revealing the vulnerability of single-point contact sensors to complex natural interferences. This finding indicates that the current fusion strategy remains dependent on the performance of individual sensors. Considering the simultaneous deployment of multiple systems in the field, a potential improvement direction is to migrate the data fusion platform to the host computer. By fusing data from multiple contact and non-contact sensors for collaborative decision-making, the impact of single-point sensor failures can be effectively isolated. Furthermore, integrating additional non-contact sensors (e.g., optical or microwave rain sensors) and introducing advanced anomaly detection mechanisms may further enhance the system’s environmental robustness.

In summary, the proposed system achieves stable state recognition with low current consumption, providing a feasible and effective protection solution for precision measuring instruments deployed in unstaffed outdoor environments.

## 6. Conclusions

This paper presents an intelligent protection system for precision measuring instruments, which adopts a subsidence-type protective structure and integrates a conflict-oriented adaptive weight adjustment model based on D-S evidence theory for multi-sensor data fusion. The core purpose of the proposed D-S evidence fusion and adaptive weight adjustment mechanism is to improve the system’s response speed in weather transition scenarios, ensuring timely protection when rainfall occurs and avoiding prolonged protection after rain stops (e.g., 3–4 h of unnecessary protection until the sensor surface is completely dry).

Simulation results on the MATLAB R2023b platform verify that the proposed adaptive weight adjustment model significantly improves the response speed of multi-sensor fusion compared with the traditional D-S method. In the sunny-to-rainy transition, the new model shortens the decision delay from 36.3 s to 21.1 s, achieving a 41.9% faster response. In the rainy-to-sunny scenario, the model reduces the decision time from 120 min to 45 min, improving the response speed by 62.5% and effectively avoiding long-term unnecessary protection after rain stops. These results demonstrate that the proposed mechanism can effectively handle sensor conflicts and ensure timely and accurate protection decisions under typical weather transitions.

Long-term field deployment results show that the system can stably operate under complex outdoor environmental conditions, with reliable state recognition and execution performance, which can effectively protect precision measuring instruments from external interference. The system has the advantages of a simple structure and easy operation; additionally, the adoption of a soft-start drive can reduce the impact of large inrush current on the system during startup, ensuring stable operation of the system.

In view of the limitations of the current system, future work will focus on optimizing the data fusion strategy to reduce the dependence on single sensor data; at the same time, more non-contact sensors will be integrated to further improve the environmental adaptability and stability of the system and provide a more reliable protection solution for field precision measurement equipment.

## Figures and Tables

**Table 1:** Key Scenario Coverage Test.

Date	Test Scenario	Contact Weight	Output Current	Expected State	Result
2025-10-3 7:00	C → LR	0.5–>0.905	2.14A	Ascend	Valid
2025-10-3 9:00	LR → O	0.2–>0.132	0.65A	Descend	Valid
2025-10-14 18:00	O → LR	0.5–>0.88	2.23A	Ascend	Valid
2025-10-14 20:00	LR → F	0.2–>0.128	0.77A	Descend	Valid
2025-10-18 10:00	O → LR	0.5–>0.932	2.12A	Ascend	Valid
2025-10-18 12:00	LR → O	0.2–>0.134	0.72A	Descend	Valid

## Data Availability

The original contributions presented in this study are included in the article. Further inquiries can be directed to the corresponding authors.
